# The *Viral Hemorrhagic Septicemia Virus* (VHSV) Markers of Virulence in Rainbow Trout (*Oncorhynchus mykiss*)

**DOI:** 10.3389/fmicb.2020.574231

**Published:** 2020-10-20

**Authors:** Laury Baillon, Emilie Mérour, Joëlle Cabon, Lénaïg Louboutin, Estelle Vigouroux, Anna Luiza Farias Alencar, Argelia Cuenca, Yannick Blanchard, Niels Jørgen Olesen, Valentina Panzarin, Thierry Morin, Michel Brémont, Stéphane Biacchesi

**Affiliations:** ^1^Virologie et Immunologie Moléculaires (VIM), Université Paris-Saclay, Institut National de Recherche pour l’Agriculture, l’Alimentation et l’Environnement (INRAE), Université de Versailles Saint-Quentin-en-Yvelines, Jouy-en-Josas, France; ^2^ANSES, Laboratoire de Ploufragan-Plouzané-Niort, Unité Pathologies Virales des Poissons, Plouzané, France; ^3^Unit for Fish and Shellfish Diseases, EURL for Fish and Crustacean Diseases, National Institute of Aquatic Resources, Technical University of Denmark (DTU), Kongens Lyngby, Denmark; ^4^ANSES, Laboratoire de Ploufragan-Plouzané-Niort, Unité Génétique Virale et Biosécurité, Ploufragan, France; ^5^Division of Comparative Biomedical Sciences, Istituto Zooprofilattico Sperimentale delle Venezie (IZSVe), Padua, Italy

**Keywords:** novirhabdovirus, *viral hemorrhagic septicemia virus*, VHSV, rainbow trout, virulence markers

## Abstract

*Viral hemorrhagic septicemia virus* (VHSV) is a highly contagious virus leading to high mortality in a large panel of freshwater and marine fish species. VHSV isolates originating from marine fish show low pathogenicity in rainbow trout. The analysis of several nearly complete genome sequences from marine and freshwater isolates displaying varying levels of virulence in rainbow trout suggested that only a limited number of amino acid residues might be involved in regulating the level of virulence. Based on a recent analysis of 55 VHSV strains, which were entirely sequenced and phenotyped *in vivo* in rainbow trout, several amino acid changes putatively involved in virulence were identified. In the present study, these amino acid changes were introduced, alone or in combination, in a highly-virulent VHSV 23–75 genome backbone by reverse genetics. A total of 35 recombinant VHSV variants were recovered and characterized for virulence in trout by bath immersion. Results confirmed the important role of the NV protein (R116S) and highlighted a major contribution of the nucleoprotein N (K46G and A241E) in regulating virulence. Single amino acid changes in these two proteins drastically affect virus pathogenicity in rainbow trout. This is particularly intriguing for the N variant (K46G) which is unable to establish an active infection in the fins of infected trout, the main portal of entry of VHSV in this species, allowing further spread in its host. In addition, salmonid cell lines were selected to assess the kinetics of replication and cytopathic effect of recombinant VHSV and discriminate virulent and avirulent variants. In conclusion, three major virulence markers were identified in the NV and N proteins. These markers explain almost all phenotypes (92.7%) observed in trout for the 55 VHSV strains analyzed in the present study and herein used for the backward validation of virulence markers. The identification of VHSV specific virulence markers in this species is of importance both to predict the *in vivo* phenotype of viral isolates with targeted diagnostic tests and to improve prophylactic methods such as the development of safer live-attenuated vaccines.

## Introduction

*Viral hemorrhagic septicemia* (VHS) is a very contagious viral disease leading to high mortality in young cultured and wild fish worldwide, and is listed as notifiable by the World Organization for Animal Health (OIE) (Skall et al., 2005; Walker and Winton, 2010). VHS is one of the fish viral diseases considered by the OIE as a serious economic and social threat for fish farms with significant environmental impact on natural resources and endangered fish species. VHS is an acute systemic disease. Fish of any age are susceptible to the infection, although fry and juveniles are the most sensitive (Wolf, 1988; Anonymous, 2019). Mortality varies depending on several environmental and physiological factors such as fish age, rearing condition, stress, fish species, virus strain, water temperature. The disease in trout is most severe at 10°C and rare at temperatures above 15°C. The virus reservoirs are infected domestic or wild fish and survivor fish of epizootic outbreaks that can become long-term carriers (Wolf, 1988; Anonymous, 2019). Transmission primarily occurs horizontally through contaminated water by direct excretion of virus from infected fish. *Viral hemorrhagic septicemia virus* (VHSV) is thought to infect fish through the gills or possibly through wounds on the skin. However, fins were shown to be the main portal of entry for a closely-related virus, the *Infectious Hematopoietic Necrosis Virus* (IHNV) (Harmache et al., 2006).

*Viral hemorrhagic septicemia* is primarily a disease of farmed rainbow trout (*Oncorhynchus mykiss*), turbot (*Scophthalmus maximus*) and Japanese flounder (*Paralichthys olivaceus*), although it is capable of infecting a broader range of freshwater and marine species (Anonymous, 2019). To date, VHSV has been isolated from more than 82 different freshwater and marine species throughout the Northern Hemisphere, including North America, Asia, and Europe (Skall et al., 2005; Jonstrup et al., 2009; Kim and Faisal, 2011). The significant prevalence of VHSV in a large number of asymptomatic marine fish species that represent an extensive potential reservoir might lead to the emergence of new highly-virulent strains for domestic fish (Kurath and Winton, 2011; Snow, 2011), thus threatening aquaculture industry. Two events illustrate these concerns well. In a VHS outbreak in marine rainbow trout farms that occurred in Norway in 2007, the sequence analysis of the VHSV isolate clearly showed that it was closely related to marine strains which were not previously considered to be pathogenic for rainbow trout, highlighting VHSV’s capacity for adaptation (Dale et al., 2009). Another example of the adaptable nature of VHSV was demonstrated by mass mortalities in 31 freshwater fish species recorded in the 2000s in the Great Lakes region of North America where no VHSV was previously reported (Kim and Faisal, 2011).

VHSV belongs to the family *Rhabdoviridae* within the order *Mononegavirales*. The virion is enveloped with a typical bullet-shaped morphology. The genome consists of a non-segmented negative-sense single-stranded RNA molecule of about 11 kilobases which encodes six proteins in the order 3′-N-P-M-G-NV-L-5′ (Schutze et al., 1999). The viral RNA is tightly encapsidated with a nucleoprotein (N), a polymerase-associated phosphoprotein (P) and the large RNA-dependent RNA polymerase (L) to form the helical ribonucleoprotein complex (RNP). The matrix protein (M) interacts with the RNP and the viral envelope and is involved in the budding step. Finally, the viral surface glycoprotein (G) is implicated in the entry step and thus represents the unique target for neutralizing and protective antibodies (Lorenzen et al., 1990; Bearzotti et al., 1995). In contrast to other rhabdoviruses, the VHSV genome possesses an additional gene, localized between the G and L genes, that encodes a small non-structural NV (Non-Virion) protein (Kurath et al., 1997). Due to the presence of the NV gene, VHSV was classified in the genus *Novirhabdovirus* together with IHNV. Using reverse genetics systems, it was demonstrated that the NV protein is essential for virus pathogenicity in rainbow trout (Thoulouze et al., 2004; Biacchesi et al., 2010), yellow perch (*Perca flavescens*) (Ammayappan et al., 2011) and Japanese olive flounder (Kim et al., 2011). The NV protein functions as inhibitor of the innate immune response by targeting cellular components of the RIG-I pathway that control the induction of interferon expression (Biacchesi et al., 2017).

Phylogenetic analyses based on the glycoprotein (G) and nucleoprotein (N) genes of VHSV led to the identification of four genotypes (Benmansour et al., 1997; Einer-Jensen et al., 2004; Snow et al., 2004) that reflect the geographic distribution of viral isolates. Isolates belonging to VHSV genotypes I, II, and III are present in continental Europe, the North Atlantic Ocean, the Baltic Sea, the North Sea and waters around Scotland. Genotype IV consists of isolates from North America and Asia. Moreover, the host range and the virulence appear, at least to some extent, to be linked to the genotype. VHSV isolates originating from marine fish (genotypes Ib, II, and III) show low pathogenicity in rainbow trout, although some of these isolates are pathogenic for turbot and herring (Skall et al., 2004; Ito et al., 2018). Similarly, VHSV isolates belonging to genotype IV have low virulence in trout (Emmenegger et al., 2013). The analysis of a few nearly complete genome sequences from marine and freshwater isolates displaying variable levels of virulence to rainbow trout suggested that only a limited number of amino acid residues might be virulence determinants (Betts and Stone, 2000; Campbell et al., 2009; Ito et al., 2018). Interestingly, molecular analysis provides evidence that the marine environment is the original reservoir of VHSV and that species barrier jumps may have occurred several times, mainly from marine fish to trout (Einer-Jensen et al., 2004; Kurath and Winton, 2011; Schonherz et al., 2018).

In a recent study by Panzarin et al. (2020), 55 VHSV isolates covering all European genotypes were selected based on field records and host of origin, and were characterized for their virulence phenotype in juvenile rainbow trout by bath immersion. All viral isolates were also subject to whole genome sequencing (WGS) and phylogenetic reconstruction based on protein coding regions. An extensive association analysis was then performed by combining virulence and sequence data to identify molecular markers putatively involved in VHSV virulence. This analysis identified 38 single amino acid polymorphisms (SAPs) dispersed over the coding regions of the genome, which could potentially modulate pathogenicity in rainbow trout and serve as molecular markers for VHSV virulence. A previous study, based on the visual inspection of a limited panel of VHSV isolates, demonstrated the reliability of this approach in pointing out a single amino acid change in the NV protein that was clearly associated with virulence in rainbow trout (Baillon et al., 2017). In the present study, 17 out of the 38 SAPs previously identified by association analysis (Panzarin et al., 2020) were evaluated, alone or in combination, by reverse genetics using the French VHSV strain 23–75 as a backbone. This virus was isolated in France in 1975 from a brown trout, and showed a highly-virulent phenotype with devastating effects on farmed rainbow trout (de Kinkelin and Le Berre, 1977; Gaudin et al., 1999). Additional 12 SAPs selected by direct comparison of closely related strains displaying contrasted phenotypes in trout were also included in this study. A total of 35 recombinant VHSV (rVHSV) variants were recovered and phenotyped in rainbow trout by bath immersion. This work identified the major role of the nucleoprotein and the non-virion protein in the virulence of VHSV strains.

## Materials and Methods

### Ethics Statement

All animal studies were carried out in strict accordance with the European guidelines and recommendations on animal experimentation and welfare (European Union directive 2010/63). All animal experiment procedures were approved by the local ethics committee on animal experimentation (COMETHEA INRA no. 45 and ANSES/ENVA/UPC no. 16) and were authorized by the Ministère de l’Éducation nationale, de l’Enseignement supérieur et de la Recherche under the numbers: APAFIS#2545-2015121515466368 v1, APAFIS#2016053117453469 and 08/04/14-10. To minimize animal suffering and distress, all manipulations were carried out under light anesthesia. Anesthesia was performed by bath immersion with 0.3 mL/L of 2-phenoxy ethanol or 0.1 to 0.2 mL/L of a 10% eugenol [2-methoxy-4-(2-propenyl) phenol] solution. A lethal challenge typically results in acute disease characterized by exophthalmia, anemia and punctiform hemorrhages. Therefore, fish were monitored twice a day for clinical signs and survival. Upon display of typical infection symptoms, animals were humanely euthanized by bath immersion using a lethal dose of 2-phenoxy ethanol (0.8 mL/L) or 10% eugenol (2 mL/L).

### Cells and Viruses

Fathead minnow (*Pimephales promelas*) EPC, rainbow trout RTG-2, Chinook salmon (*Oncorhynchus tshawytscha*) CHSE-214 and bluegill (*Lepomis macrochirus*) BF-2 cells were maintained in Glasgow’s modified Eagle’s medium-HEPES 25 mM medium supplemented with 10% fetal bovine serum and 2 mM L-glutamine and incubated at 24°C for EPC cells and at 20°C for RTG-2, CHSE-214 and BF-2 cells.

rVHSV were propagated in monolayer cultures of EPC cells at 15°C as previously described (Fijan et al., 1983; Biacchesi et al., 2010). Virus titers were determined by plaque assay on EPC cells under an agarose overlay (0.35% agarose in Glasgow’s modified Eagle’s medium-HEPES 25 mM medium supplemented with 2% fetal bovine serum and 2 mM L-glutamine). At 3–4 days postinfection, cell monolayers were fixed with 10% formol and stained with crystal violet. Recombinant vaccinia virus expressing the T7 RNA polymerase, vTF7-3 (Fuerst et al., 1986), was kindly provided by B. Moss (National Institutes of Health, Bethesda, MD, United States). For multiple growth kinetics, EPC, RTG-2, CHSE-214, and BF-2 cells were infected at an MOI of 0.01 PFU per cell with variant rVHSV viruses. Supernatant aliquots (0.2 mL out of a total medium volume of 2 mL per well) were taken at different times postinfection and replaced by an equivalent volume of fresh medium. The samples were flash-frozen and analyzed later by plaque assay.

### Construction of rVHSV Variant cDNA and Recombinant Virus Recovery

The plasmid pVHSV, which contains the complete consensus antigenomic sequence of VHSV 23–75 (Biacchesi et al., 2010), was modified by site directed mutagenesis using the QuickChange Multi Site-Directed Mutagenesis Kit (Stratagene). Recombinant viruses were named according to the viral protein together with the position and the amino acid change. Specific primers were designed based on the exact codon found in variant strain sequences (Panzarin et al., 2020) and are available upon request. The mutagenesis reaction was performed on VHSV fragments subcloned in pJET1.2 plasmid (CloneJET PCR cloning kit; Fermentas). The mutated subclones were then cloned back into pVHSV. rVHSV-N K46G expressing the *Renilla luciferase* gene was obtained from a previously described construct, pVHSV-Tomato expressing the Tomato fluorescent protein from an additional cassette inserted between N and P genes (Biacchesi et al., 2010). The Tomato gene was replaced by the one encoding *Renilla luciferase* and the cassette was then introduced in the pVHSV-N K46G backbone using the unique *Psi*I restriction enzyme site. The recovery of rVHSV was carried out as described previously (Biacchesi et al., 2010). Approximately 4 × 10^6^ EPC cells per well were grown in six-well plates and infected with the recombinant vaccinia virus vTF7-3 expressing the T7 RNA polymerase at a multiplicity of infection of 5. After 1 h adsorption at 37°C, cells were washed twice and transfected with a plasmid mixture containing 0.25 μg of pT7-N, 0.2 μg of pT7-P, 0.2 μg of pT7-L and 1 μg of the pVHSV full-length cDNA construct using JetPEI transfection reagent (Polyplus Transfection) according to the supplier’s instructions. The cells were incubated for 6 h at 37°C and then shifted at 14°C for 6 days. Subsequently, the cells were suspended by scratching the plates with a rubber policeman and then subject to two cycles of freezing and thawing. The cell culture supernatant (called P0 supernatant) was clarified by centrifugation at 6,000 × *g* for 10 min in a microcentrifuge at 4°C and used to inoculate fresh EPC cell monolayers in 24-well plates at 14°C. The recombinant virus genome sequence was verified by RT-PCR amplification and nucleotide sequencing (Eurofins Genomics) was performed on viral RNA extracted from infected-cell supernatants after three passages on EPC cells (Biacchesi et al., 2000a).

### Experimental Fish Infection and Bioluminescence Imaging

Virus-free juvenile INRA synthetic strain (25–50, as indicated in the figure legends), a domesticated strain maintained in the experimental facilities, or ANSES strain (80–100 in duplicate or triplicate tanks, as indicated in the figure legends) of rainbow trout (mean weight, 0.68–103 g) were infected by immersion in tanks filled with 3–10 L of freshwater with variant rVHSV viruses (final titer, 5 × 10^4^ PFU/mL) for 2 h at 10°C. Tanks were then filled up to 30 L with freshwater and the flow was turned on (open water system, 10 L/hour). Controls were fish mock infected with cell culture medium under the same conditions. Mortalities were recorded every day over a period of 23–40 days. Juvenile trout were also infected by intraperitoneal injection with 10^6^ PFU/fish under anesthesia. For a few selected rVHSV variants, surviving trout were challenged by immersion at days 33–35 with the virulent rVHSV as described above (final titer, 5 × 10^4^ PFU/mL). Mortality was recorded daily for approximately 2–3 weeks. Virus isolation was performed on a subset of dead fish. Whole fish were homogenized in a mortar with a pestle and sea sand in Glasgow’s modified Eagle’s medium-HEPES 25 mM medium containing penicillin (200 IU/mL), streptomycin (0.2 mg/mL), kanamycin (0.2 mg/mL) and amphotericin B (2.5 mg/mL). After centrifugation at 3,000 × *g* for 15 min at 4°C, the supernatants were used to inoculate EPC cells as described previously, and the virus titer in each sample was determined by plaque assay.

For *in vivo* bioluminescence imaging, trout were infected by immersion as described above. At 24 h and 72 h post infection, 4–8 fish were randomly transferred in a small tank with water containing Enduren live cell substrate (Promega) (1/10,000 dilution) as previously published (Harmache et al., 2006). Two hours later, anesthetized fish were subjected to imaging using an IVIS 200 imaging system (PerkinElmer). Living Image software (version 4.0, PerkinElmer) was used both to acquire bioluminescent and photographic images.

### Phylogenetic Analysis

The phylogenetic tree used in this study was previously developed by Panzarin et al. (2020) in a study aiming at identifying candidate VHSV virulence markers by statistical association analysis. In brief, 55 VHSV genomes were aligned using MAFFT v7.388 (Katoh et al., 2002) with default settings. Genome termini, intergenic regions, and stop codons were removed. This generated a matrix of 10,296 nt encompassing coding region only. Phylogenetic analysis was carried out using RAxML v. 8 (Stamatakis, 2014) with the GTR + G model, and all free parameters estimated by the program, with 1,000 rapid bootstrap inferences and search for the best-scoring ML tree.

### Statistical Analyses

Data analyses were performed using GraphPad Prism. Kinetics of survival between wild-type and variant rVHSV groups within each experiment were analyzed by Kaplan–Meier estimation of survival curves, followed by comparison of the survival curves by Log-rank test (Mantel–Cox) for significant differences. Kinetics of survival curves were assigned to statistical groups. Values within a column that share a common letter are not significantly different; values with different letters are significantly different (*p* < 0.05). The median survival, the time at which fractional survival equals 50%, was also calculated.

## Results

### Recovery of rVHSV Variants

Unique restriction enzyme sites found in VHSV 23–75 infectious cDNA (Biacchesi et al., 2010) were used to subclone VHSV genomic fragments in which nucleotide substitutions were inserted by site-directed mutagenesis. After sequencing, these mutated fragments were transferred back in VHSV 23–75 cDNA backbone. The recombinant viruses were readily recovered on EPC cells as previously described (Biacchesi et al., 2010). A total of 35 rVHSV variants, containing single or multiple substitutions, were produced: 4 variants in N, 3 variants in P, 3 variants in M, 5 variants in G, 10 variants in NV, 4 variants in L and 6 variants with 2–3 substitutions in one or two different viral proteins (Tables 1, 2). Each recombinant virus was amplified by two or three passages on EPC cells and the viral stock was titrated by plaque assay on EPC cells. Almost all variants reached high titers between 1 × 10^8^ to 1 × 10^9^ PFU/mL similar to that obtained by rVHSV (1 × 10^9^ PFU/mL). In contrast, 3 variants in M (T195A, S197P and T195A/S197P) and 1 variant in G (T135A) reached somewhat lower titers between 3 × 10^6^ to 9 × 10^7^ PFU/mL. The nucleotide sequencing confirmed that except for targeted substitution(s), no other incidental mutations were introduced in the rVHSV variant genomes.

**TABLE 1:** Characteristics of rVHSV 23–75 variants with a single amino acid substitution *in vitro* in cell culture and *in vivo* in rainbow trout.

Protein (AA position)^1^	Viral titer^2^ (PFU/mL)	% of mortality at 20 days PI^3^	% of mortality at 30 days PI^3^	% of mortality at 26 days PC^4^
**rVHSV**	**1 × 10^9^**	68–100*	75–100*	
**N protein**				
G42S	2 × 10^8^	68	69	
K46G	3 × 10^8^	0	0	78
A241E	2 × 10^8^	24	25	
G392E	2 × 10^8^	70	78	
**P protein**				
R23K	7 × 10^8^	96	–	
L70R	2 × 10^8^	98	–	
A131S	3 × 10^8^	100	–	
**M protein**				
T195A	3 × 10^6^	90	–	
S197P	6 × 10^6^	86	–	
W201R	1 × 10^9^	95	99	
**G protein**				
D51E	7 × 10^8^	93	–	
T135A	9 × 10^7^	57	–	
T277A	2 × 10^8^	94	94	
R283K	8 × 10^8^	95	–	
V290I	1 × 10^9^	94	–	
**NV protein**				
A2T	2 × 10^8^	100	100	
L15F	8 × 10^8^	84	89	
V45M	2 × 10^8^	96	96	
H67Y	1 × 10^8^	96	96	
V104I	8 × 10^8^	71	72	
L113V	1 × 10^9^	73	77	
T115I	2 × 10^8^	67	73	
R116S	1 × 10^8^	16	20	9
R116N	1 × 10^8^	94	94	
L120P	1 × 10^8^	89	91	
**L protein**				
E149G	1 × 10^8^	100	100	
F1012I	1 × 10^9^	71–100	86–100	
M1313T	3 × 10^8^	92	96	
A1732T	8 × 10^8^	100	100	

**FIGURE 1:**
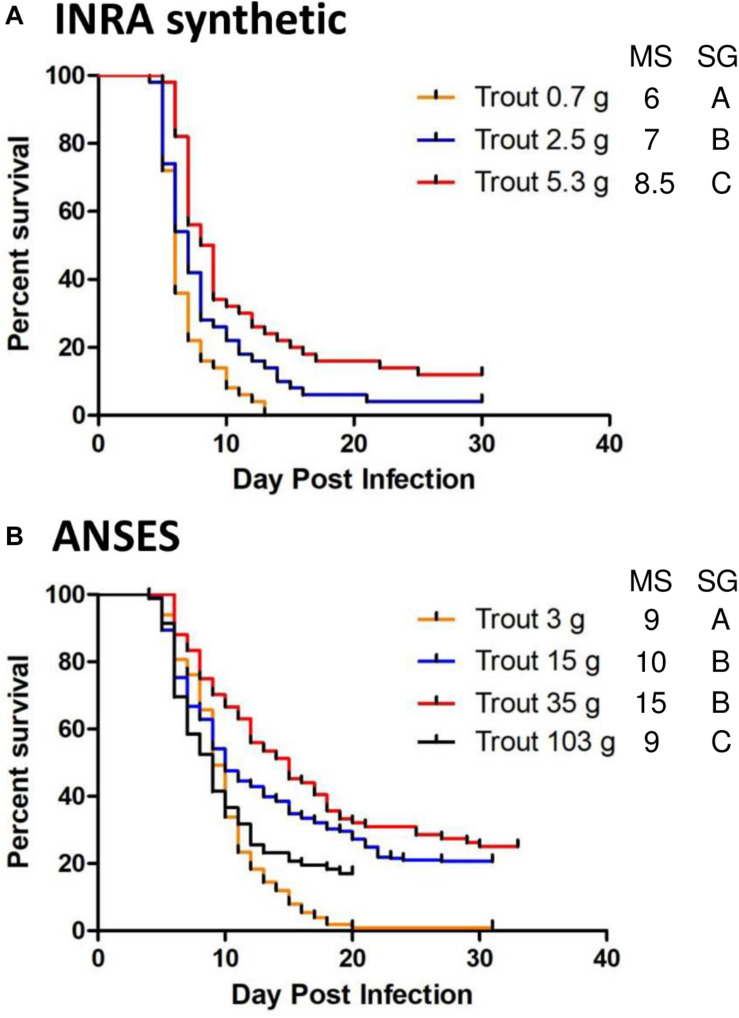
Weight-dependent susceptibility of rainbow trout against a lethal infection. Virus-free juvenile rainbow trout INRA synthetic strain (*n* = 50 per group) **(A)** or ANSES strain (*n* = 80 per group in duplicate; means are shown) **(B)** of different body weights (mean weights, from 0.7 to 103 g) were infected by immersion with rVHSV 23–75 (final titer, 5 × 10^4^ PFU/mL) for 2 h at 10°C. Mortality was recorded daily and is presented as percent of survival. Median survival (MS) and statistical grouping (SG; *p* < 0.05) are shown on the right of each graph.

*^1^Nature and position of the amino acid substitution in rVHSV 23–75 backbone. ^2^Virus titer calculated from titration by plaque assay in EPC cells. ^3^Cumulative percent of mortality recorded at 20 and 30 days post infection (PI). ^4^Cumulative percent of mortality recorded at day 26 post challenge (PC) of surviving fish infected with indicated rVHSV variants and challenged with rVHSV 23–75 4 weeks later. ^∗^Range of cumulative percent of mortality for rVHSV 23–75 in all trials irrespective of the fish strain and mean body weight and recorded at 20 and 30 days post infection, respectively (see Figure 1).*

**TABLE 2:** Characteristics of rVHSV 23–75 variants with multiple amino acid substitutions *in vitro* in cell culture and *in vivo* in rainbow trout.

Protein (AA position)^1^	Viral titer^2^ (PFU/mL)	% of mortality at 20 days PI^3^	% of mortality at 30 days PI^3^	% of mortality at 26 days PC^4^
**rVHSV**	**1 × 10^9^**	68–100*	75–100*	
**Multiple changes**				
N-K46G/G392E	2 × 10^8^	0	0	98
N-G392E/NV-R116S	8 × 10^8^	40	44	10
N-G392E/L-F1012I	9 × 10^8^	46–64	48–92	4
M-T195A/S197P	4 × 10^6^	80	_	
NV-A2T/R116N	4 × 10^8^	75	77	
NV-L15F/T115I/L120P	8 × 10^8^	65	70	

*^1^Nature and position of the amino acid substitutions in rVHSV 23–75 backbone. *^2^Virus titer calculated from titration by plaque assay in EPC cells. ^3^Cumulative percent of mortality recorded at 20 and 30 days post infection (PI). ^4^Cumulative percent of mortality recorded at day 26 post challenge (PC) of surviving fish infected with indicated rVHSV variants and challenged with rVHSV 23–75 4 weeks later. ^∗^Range of cumulative percent of mortality for rVHSV 23–75 in all trials irrespective of the fish strain and mean body weight and recorded at 20 and 30 days post infection, respectively (Figure 1)*.*

### Pathogenicity of rVHSV Variants in Juvenile Trout

The pathogenicity of rVHSV variants was evaluated by infecting juvenile rainbow trout by bath immersion and recording the virus-induced mortality rate from 23 to 40 days post infection. During this study two strains of rainbow trout, namely INRA synthetic and ANSES strains, were used in the trials with varying body weights. The weight-dependent susceptibility of each rainbow trout strain against a lethal infection was first analyzed for the wild-type rVHSV. A significant increase in survival (*p* < 0.05) was observed with higher body weight, although both trout strains at each tested body weight remained highly susceptible to the disease (Figure 1). This phenomenon was more evident for INRA synthetic compared to ANSES strain for which no clear correlation was observed between fish weight and the cumulative percent of mortality (CPM). For INRA synthetic strain, the CPM were from 88 to 100% within the body weight range that was used in the different experiments (from 0.7 to 5.3 g). For the ANSES strain, a larger range of body weights was used, from 3 to 103 g, but fish remained highly susceptible to rVHSV infection at any size, with CPM ranging from 75 to 100%. In all of the following infection experiments, rVHSV variants were compared to rVHSV wild-type control infection performed concomitantly and using similar fish lots.

#### rVHSV-N Variants

**FIGURE 2:**
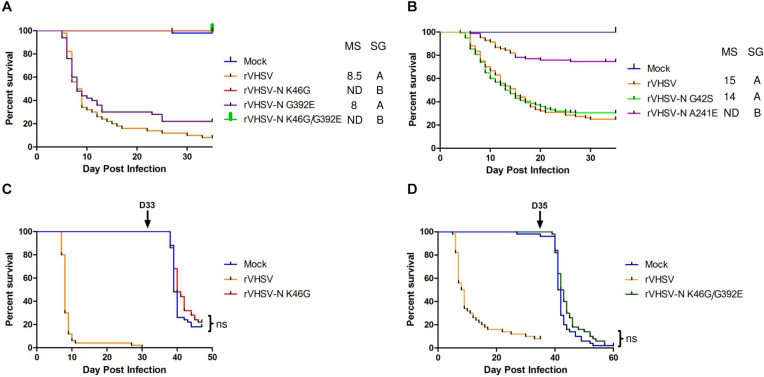
Phenotype in trout of rVHSV bearing amino acid changes in the N protein. **(A,B)** Juvenile trout [mean weights, 5.3 g (*n* = 50 per group) and 35 g (*n* = 80 per group) for **A,B**, respectively] were infected by bath immersion with 5 × 10^4^ PFU/mL of each of the indicated variants. rVHSV is a wild-type recombinant virus derived from the French strain 23–75. Mortality was recorded daily and is presented as percent of survival. Mock, non-infected trout. Median survival (MS; ND for undefined) and statistical grouping (SG; *p* < 0.001) are shown on the right of each graph. **(C,D)** Juvenile trout (mean weights, 0.5 and 2.5 g for **C,D**, respectively, and *n* = 50 per group) were infected by bath immersion with 5 × 10^4^ PFU/mL of each of the indicated N variants and the rVHSV as control. Surviving trout in both groups infected with the N variants were then challenged by bath immersion at day 33 or 35 (arrow), respectively, with the virulent rVHSV 23–75 strain. The mock-infected groups were infected in parallel and serve as positive controls of the challenge infection. Mortality was recorded daily for approximately 2–3 additional weeks. ns for non-significant (*p* > 0.05).

Four SAPs in the N protein were tested in juvenile rainbow trout alone or in combination: G42S, K46G, A241E, and G392E. As shown in Figure 2A, the G392E variant displayed similar kinetics of induced mortality compared to rVHSV control with a mortality starting at day 5 and reaching almost 50% at day 8 post infection. However, the final CPM was slightly lower for rVHSV-N G392E (78%) compared to rVHSV (90%). The G42S substitution had no effect on virulence (Figure 2B). In contrast, rVHSV-N A241E was attenuated *in vivo* (*p* < 0.001), inducing only 25% of CPM at day 35 compared to 75% for rVHSV (Figure 2B). Interestingly, no mortality was recorded for both viruses harboring K46G substitution (alone or in combination with G392E) during the entire experimental period, pointing out that the N protein plays an important role as a VHSV virulence determinant in rainbow trout (Figure 2A). Surviving fish from the initial infection trial with rVHSV-N K46G (Figure 2C) and rVHSV-N K46G/G392E (Figure 2D) were challenged with the highly-virulent rVHSV 23–75 strain at days 33 and 35 post primary infection, respectively. The mock-infected fish control groups were highly susceptible to rVHSV challenge with CPM of 82% (14 days post challenge) and 98% (25 days post challenge). Surprisingly, in both groups primarily infected with K46G variants, no significant differences (*p* > 0.05) in fish mortality were observed compared to the mock-infected fish group. For both viruses, the CPM reached 78 and 98% at the end of the challenge experiment, meaning that the initial infection by K46G variants did not induced any immune protection.

**FIGURE 3:**
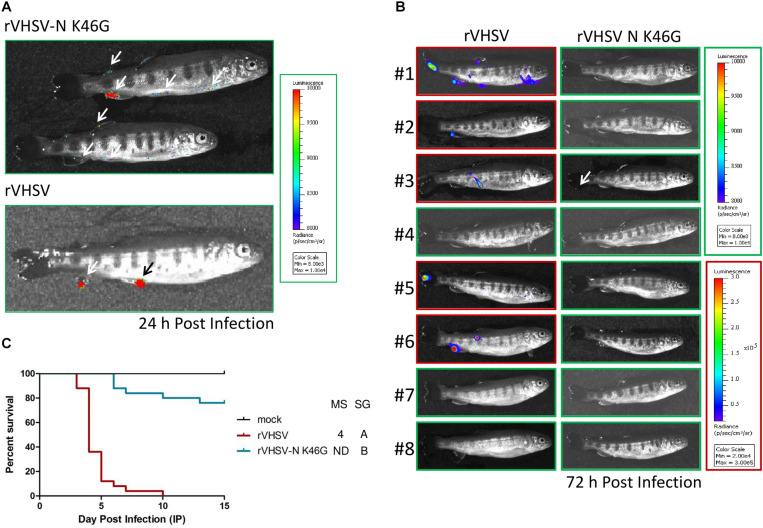
Kinetics of rVHSV-N K46G variant infection in trout. **(A,B)** Juvenile trout (mean weights, 1.4 g; *n* = 25 per group) were infected by bath immersion with 5 × 10^4^ PFU/mL of rVHSV 23–75 strain or rVHSV-N K46G variant both expressing the Renilla reporter gene from an additional cassette inserted between N and P genes. At 24 h (A) and 72 h **(B)** post infection, infected fish, randomly harvested, were analyzed by bioluminescence imaging and luminescence radiance measurements are indicated by a color scale. **(A)** Positive signals detected in rVHSV-N K46G- and rVHSV-infected trout are shown by arrows. **(B)** Images of luminescence radiance measurements are bordered in red for positive fish and in green for negative fish for which the sensitivity was increased in order to detect some signal. A white arrow indicates a spot of virus replication observed in one rVHSV-N K46G infected trout. **(C)** Virus free juvenile trout (mean weights, 3.4 g; *n* = 25 per group) were infected by intraperitoneal (IP) injection with 10^6^ PFU/fish. Mortality was recorded daily for 15 days. Mock, cell medium-injected trout. Median survival (MS; ND for undefined) and statistical grouping (SG; *p* < 0.001) are shown on the right of each graph.

In order to investigate whether these K46G variants were able to actively infect juvenile rainbow trout, a non-invasive bioluminescence approach was developed so that replication in live fish could be followed after infection, as previously described for IHNV (Harmache et al., 2006). Thus, an additional expression cassette encoding the *Renilla luciferase* was inserted in rVHSV and in rVHSV-N K46G between the N and P genes. Juvenile trout were then infected by bath immersion with both viruses. At two time points post infection, the fish were randomly sampled and transferred in a smaller tank containing a water-soluble luciferase substrate. At 24 h post infection, replication of the virus was assessed in the trout fins of both groups. Two out of four sampled fish in both groups showed actively replicating viruses in fins as shown for rVHSV-N K46G-infected fish in Figure 3A. At 72 h post infection, five out of eight sampled fish were positive for rVHSV with larger infectious foci in the fins and the skin (Figure 3B). In contrast, virus replication in rVHSV-N K46G-infected fish group was not detectable at all, except for a small spot of residual luciferase activity in the tail of one fish (fish #3 in Figure 3B). This unexpected result indicates that in approximately 48 h, rVHSV-N K46G was rapidly cleared from the fins of infected fish. To ascertain that the rVHSV-N K46G variant virus has no defect in its efficiency to establish an active infection at the portal of entry in its host, 25 juvenile trout were infected by intraperitoneal injection of a high dose of virus (10^6^ PFU/fish). As shown in Figure 3C, 100% of fish injected with rVHSV died in 10 days. In contrast, in rVHSV-N K46G-infected group, first mortalities were observed at day 6 post infection (3 days later compared to rVHSV-infected fish) and CPM reached a plateau of 24%, thus demonstrating that the K46G substitution in the N protein had a substantial effect on rVHSV virulence in rainbow trout.

#### rVHSV-P, -M, and -G Variants

**FIGURE 4:**
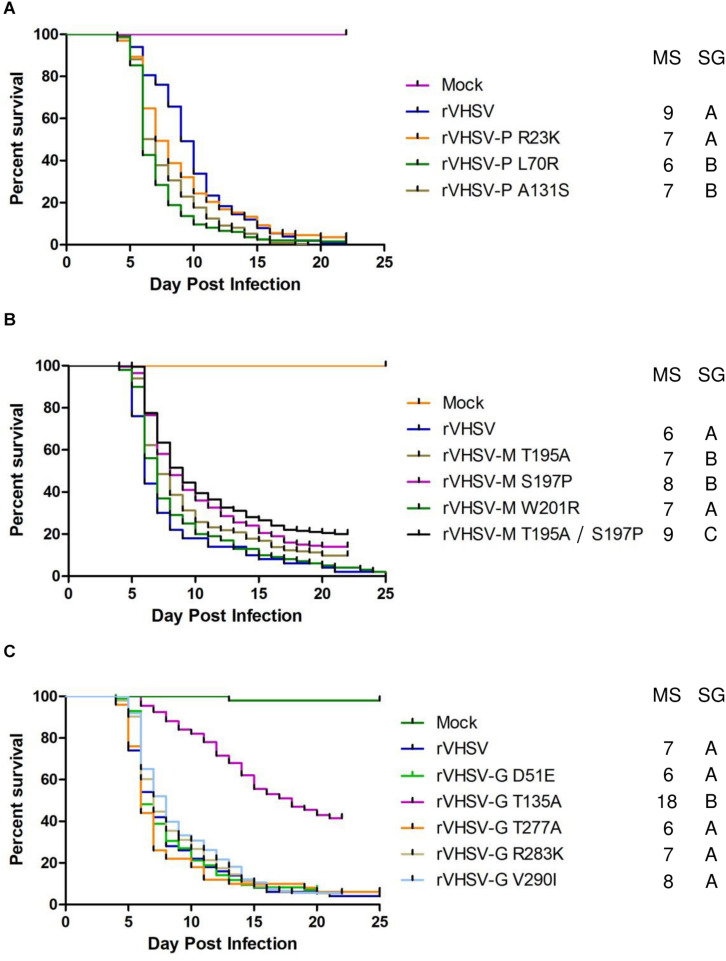
Phenotype in trout of rVHSV bearing amino acid changes in the P **(A)**, M **(B)**, and G **(C)** proteins.Juvenile trout (mean weights, 3, 1.2, and 2.5 g for **A–C**, respectively; *n* = 100 per groups in duplicate; means are shown) were infected by bath immersion with 5 × 10^4^ PFU/mL of each of the indicated variants. rVHSV is a wild-type recombinant virus derived from the French strain 23–75. Mortality was recorded daily and is presented as percent of survival. Mock, non-infected trout. Median survival (MS) and statistical grouping (SG; *p* < 0.001) are shown on the right of each graph.

A similar approach was followed in order to study putative virulence markers found in P, M, and G proteins. Three SAPs were investigated in the P and the M proteins. As shown in Figures 4A,B, all of these amino acid substitutions had no effects on final rVHSV-induced mortality (CPMs higher than 86%) even if some of them led to significant differences in the survival kinetics. This is in contrast with what was observed for T195A and S197P substitutions in the M protein that led to attenuation *in vitro* in EPC cells with roughly 2- to 3-log reduction in final viral titers (Table 1). Because both substitutions T195A and S197P are found together in several field strains with low virulence to rainbow trout, a recombinant virus harboring both substitutions, rVHSV-M T195A/S197P, was produced. This virus had a similar attenuation *in vitro* in EPC cells and was also attenuated *in vivo* in trout (80% of CPM compared to 96% of CPM for rVHSV with *p* < 0.001) (Figure 4B).

For the G envelope glycoprotein, 5 SAPs were tested in the rVHSV backbone (Figure 4C). Four of them, D51E, T277A, R283K, and V290I, had no effects on rVHSV virulence (*p* > 0.05), while the T135A substitution strongly attenuated rVHSV virulence in trout (*p* < 0.001) with CPM only reaching 57% compared to 96% for rVHSV, at day 20 post infection. The T135A substitution also led to a somewhat lower replication in EPC cells with final viral titers reduced by roughly 1 log (Table 1). All together these results strongly suggest that the G protein is involved in VHSV virulence in rainbow trout.

#### rVHSV-NV Variants

**FIGURE 5:**
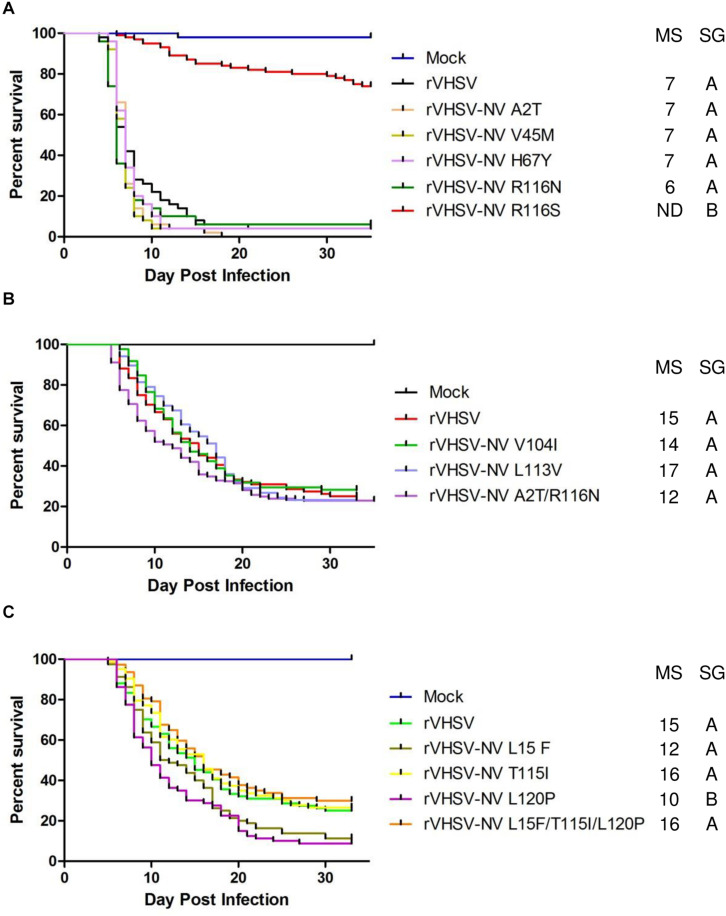
Phenotype in trout of rVHSV bearing amino acid changes in the NV protein. **(A–C)** Juvenile trout (mean weights, 0.7 g and *n* = 50 per groups for A and mean weights, 35 g and *n* = 80 per groups for **B,C**) were infected by bath immersion with 5 × 10^4^ PFU/mL of each of the indicated variants. rVHSV is a wild-type recombinant virus derived from the French strain 23–75. Mortality was recorded daily and is presented as percent of survival. Mock, non-infected trout. Median survival (MS) and statistical grouping (SG; *p* < 0.001) are shown on the right of each graph.

As previously demonstrated, the NV protein is clearly involved in VHSV virulence, with a specific amino acid substitution in position 116 (R116S) shown to regulate pathogenicity (Baillon et al., 2017). In the present study, we confirmed that R116S substitution had a highly attenuating effect (*p* < 0.001) on rVHSV virulence in rainbow trout (20% of CPM versus 98% for rVHSV; Figure 5A). In contrast, the R116N substitution had no effect on rVHSV virulence (*p* > 0.05), although it was found in several low virulent VHSV field strains in trout (Baillon et al., 2017). Eight new SAPs were analyzed: A2T, L15F, V45M, H67Y, V104I, L113V, T115I, and L120P (Table 1 and Figures 5A–C). None of them had an attenuating effect on rVHSV virulence. In fact, L120P substitution even had a significant enhancing effect (*p* < 0.01) on rVHSV virulence with CPM reaching 91% compared to 75% of CPM induced by rVHSV in trout of mean weight of 35 g. Two combinations of those substitutions were also tested since they were found co-occurring in low virulence field strains: A2T/R116N and L15F/T115I/L120P (Table 2). For both combinations, no differences were observed (*p* > 0.05), although recombinant virus harboring L120P substitution alone was somewhat more virulent. It is possible that SAPs combinations other than those herein tested might have an effect on rVHSV *in vivo* virulence.

#### rVHSV-L Variants

**FIGURE 6:**
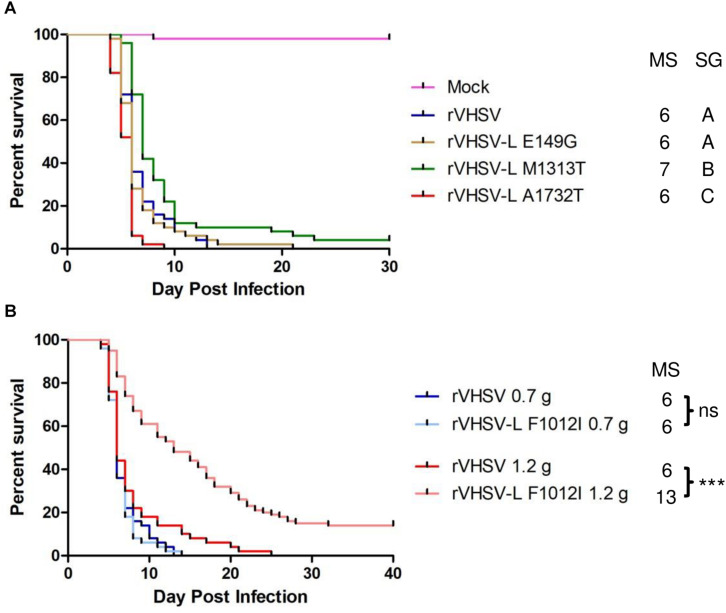
Phenotype in trout of rVHSV bearing amino acid changes in the L protein and effect of fish size on this phenotype. **(A)** Juvenile trout (mean weights, 0.7 g and *n* = 50 per groups) were infected by bath immersion with 5 × 10^4^ PFU/mL of each of the indicated variants. rVHSV is a wild-type recombinant virus derived from the French strain 23–75. Mortality was recorded daily and is presented as percent of survival. Mock, non-infected trout. Median survival (MS) and statistical grouping (SG; *p* < 0.01) are shown on the right of the graph. **(B)** Two groups of 50 juvenile trout (mean weight of 0.7 and 1.2 g, respectively) were both infected by bath immersion with 5 × 10^4^ PFU/mL of rVHSV and rVHSV-L F1012I. Median survival (MS) and statistical significance [ns for non-significant (*p* > 0.05) and *** for *p* < 0.001] are shown on the right of the graph.

It was previously proposed that the L protein participates in defining the host species virulence of various VHSV strains (Kim et al., 2014). A single amino acid substitution, I1012F, in the L protein was sufficient to render a marine VHSV strain able to replicate and induce a cytopathic effect (CPE) in trout gill epithelial cells. In the present study, 4 SAPs putatively involved in the virulence were analyzed, including the F1012I substitution (Table 1). All rVHSV-L variants were as virulent as the rVHSV 23–75 in juvenile trout (mean weight of 0.7 g) (Figures 6A,B), although two of them led to significant differences in the survival kinetics. In the case of rVHSV-L F1012I, a slower kinetic of mortality was observed in larger trout (mean weight of 1.2 g) with a final CPM of 86% versus 100% of the rVHSV at 40 days post infection (Figure 6B), indicating that this particular substitution in the L protein could be linked, at least partially, to VHSV strain virulence in trout (*p* < 0.001).

#### rVHSV Harboring Combinations of SAPs

**FIGURE 7:**
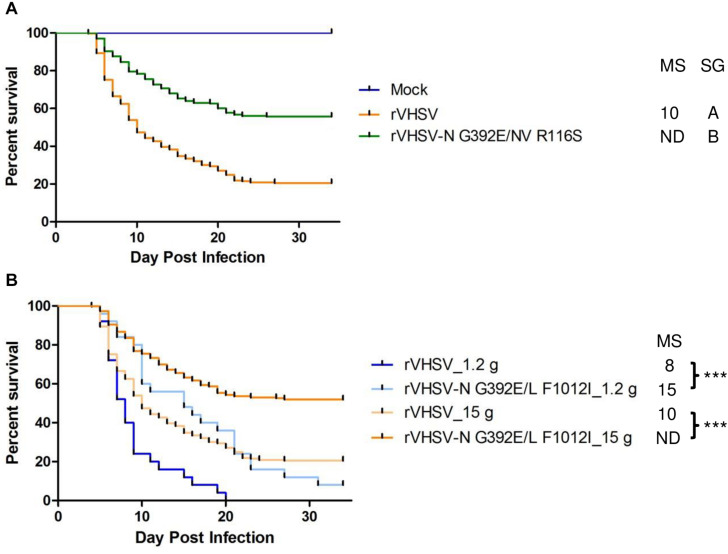
Phenotype in trout of rVHSV bearing amino acid changes in two different viral proteins and effect of fish size on this phenotype. **(A)** Juvenile trout (mean weights, 1.2 g and *n* = 100 per groups in triplicate; means are shown) were infected by bath immersion with 5 × 10^4^ PFU/mL of each of the indicated variants. rVHSV is a wild-type recombinant virus derived from the French strain 23–75. Mortality was recorded daily and is presented as percent of survival. Mock, non-infected trout. Median survival (MS) and statistical grouping (SG; *p* < 0.001) are shown on the right of the graph. **(B)** Two groups of 100 juvenile trout (mean weight of 1.2 and 15 g, respectively) were both infected in triplicate by bath immersion with 5 × 10^4^ PFU/mL of rVHSV and rVHSV-N G392E/L F1012I; means are shown. Median survival (MS) and statistical significance (*** for p < 0.001) are shown on the right of the graph.

Two other combinations of substitutions located in two different proteins were tested since they were observed to co-occur in the genomes of several low virulence field strains. These substitutions were found in viral proteins belonging to the viral replication complex such as N, L, and NV proteins. The NV protein has been previously shown to co-immunoprecipitate with the RNP complex and to play a role in either the replication or transcription steps (Biacchesi et al., 2000b, 2017; Thoulouze et al., 2004). rVHSV-N G392E/NV R116S was attenuated in trout (*p* < 0.001) with only 44% of induced CPM at day 30 post infection compared to 79% for the virulent rVHSV strain (Figure 7A), indicating that the addition of the G392E substitution in the N protein within the NV R116S backbone had no additional effect. In contrast, this was not the case for rVHSV-N G392E/L F1012I. Although this variant displayed similar kinetics of induced mortality compared to the single variant rVHSV-L F1012I in trout of similar body weight (Figures 6B, 7B, respectively), the attenuated phenotype of rVHSV-N G392E/L F1012I was more pronounced in larger trout (Figure 7B). Indeed, the kinetics of mortality was slower for rVHSV-N G392E/L F1012I in trout of 1.2 g (*p* < 0.001), but the final CPM recorded was quite similar to that of rVHSV 23–75 (92% versus 100%, respectively). In contrast, in larger trout of mean weight of 15 g, rVHSV-N G392E/L F1012I appeared attenuated reaching only 48% of induced mortality compared to 79% for the virulent rVHSV strain (*p* < 0.001), highlighting a potential role of these particular positions in the N and L proteins in VHSV strain virulence in trout.

### Comparison of Replication Kinetics and Induction of CPE by rVHSV Variants in Cell Culture

**FIGURE 8:**
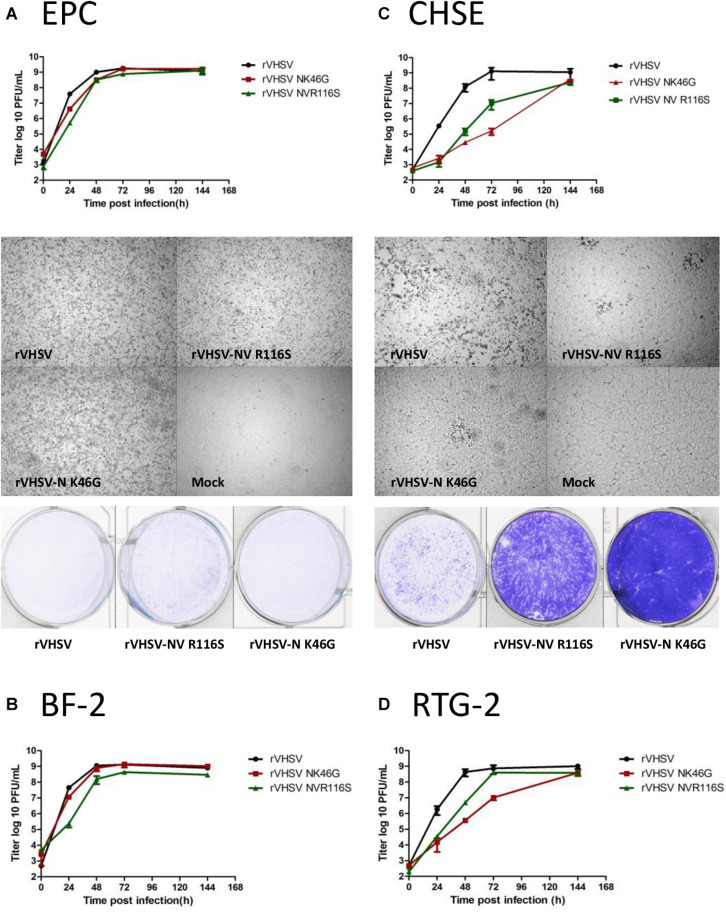
*In vitro* kinetics of replication of rVHSV-N K46G and rVHSV-NV R116S on salmonid and cyprinid cell lines. Multi-step growth curves. EPC **(A)**, CHSE 214 **(B)**, BF-2 **(C)**, and RTG-2 **(D)** cells were infected at an MOI of 0.01 with wild-type rVHSV, rVHSV-N K46G, and rVHSV-NV R116S. Supernatant aliquots were taken at the indicated time points and analyzed by plaque assay. Each time point was tested in two wells, and each virus titration was done in duplicate. Means are shown together with the calculated standard errors.Virus-induced cytopathic effect. EPC **(A)** and CHSE 214 **(B)** cell monolayers were visualized under a microscope at day 3 postinfection and then stained with crystal violet 6 days postinfection.

In order to identify a fish cell line able to discriminate between low- and high-virulence rVHSV variants, four cell lines were tested: two non-salmonid cell lines regularly used to amplify VHSV, cyprinid EPC cells (*Pimephales promelas*) and centrarchid BF-2 cells (*Lepomis macrochirus*); and two salmonid cell lines derived from the genus *Oncorhynchus*, rainbow trout RTG-2 cells and Chinook salmon CHSE-214 cells (*Oncorhynchus tshawytscha*). rVHSV-N K46G and rVHSV-NV R116S viruses were compared to wild-type rVHSV 23–75 with regards to the efficiency of multicycle replication in these four cell lines following infection with an input of 0.01 PFU per cell (Figure 8). On EPC cells, both rVHSV variants replicated with the same efficiency as rVHSV (Figure 8A). The final titers reached by both viruses were roughly 1 × 10^9^ PFU/mL. No delay was observed in the CPE appearance and all variants induced a total CPE at day 6 post infection. Thus, N K46G and NV R116S substitutions do not seem to affect viral replicative fitness *in vitro* in that cell line. A similar result was observed in BF-2 cells (Figure 8B), except that rVHSV-NV R116S did not induce a total CPE in this cell line. In contrast, in salmonid cells, CHSE-214 (Figure 8C) and RTG-2 (Figure 8D), both rVHSV NV and N variants replicated with an efficiency reduced by 2- to 3-log and 3- to 4-log, respectively, on day 2 post infection compared to that of rVHSV. However, the final titer reached by both variants was close to that of rVHSV, roughly 1 × 10^8^ versus 1 × 10^9^ PFU/mL. The slower replication kinetics, observed for both variants, were characterized by an important delay in the appearance of the virus-induced CPE. A total CPE was not reached at day 6 post infection for both variants in both salmonid cell lines. The difference between CPE induced by both NV and N variants compared to that of rVHSV was more pronounced in CHSE-214 cells. In summary, CHSE-214 cells are a good cell substrate to discriminate these low- and high-virulence rVHSV variants.

### Phylogenetic Analysis of Virulence Markers

**FIGURE 9:**
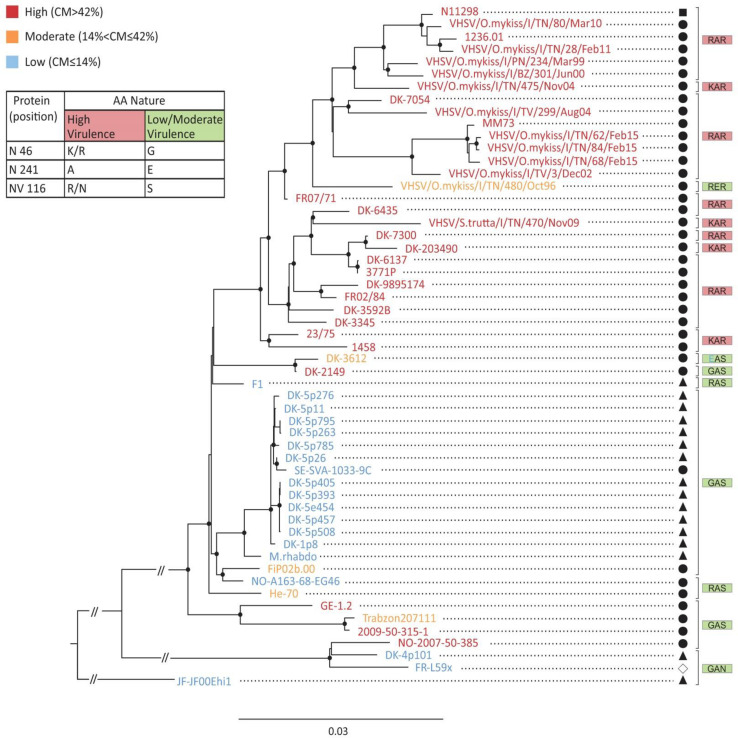
Phylogenetic tree and virulence markers. Phylogenetic relationship among VHSV strains inferred with Maximum Likelihood method using the complete protein coding sequences. VHSV isolate names are color-coded based on the following thresholds of virulence in rainbow trout: in red: mortality >42%; in orange: mortality between 42 and 14%; in blue: mortality ≤14%. The nature and position of amino acid residues involved in virulence in rainbow trout are indicated in the table legend. The 3 amino acid code for each VHSV field isolates is shown in the tree. The amino acid in blue is an amino acid substitution that was not tested in this study. Genome sequences of each VHSV strain are available in GenBank and referenced in Panzarin et al. (2020). The host origin of each VHSV isolates is indicated: ▲ marine fish species (flounder, turbot, whiting, haddock, cod, herring, sprat, plaice, and dab), 🌑 salmonids (rainbow trout and brown trout), ■ freshwater fish (pike) and ◆ catadromous fish (eel).

In a recent study by Panzarin et al. (2020), 55 VHSV isolates, covering all European genotypes and isolated from a large panel of marine and freshwater fish species, were characterized for their degree of virulence in rainbow trout and fully-sequenced. Strains were assigned to different virulence groups (high, moderate, and low), based on the cumulative mortality observed. Sequence and phenotype data were used for association analyses and phylogenetic inference to identify putative virulence markers in VHSV genome. The main VHSV virulence markers validated in the present study and listed in Figure 9, were superimposed on the phylogenetic tree generated by Panzarin et al. (2020). Two of these three markers are located in the N protein (K/R46G and A241E) and one in the NV protein (R/N116S). For each VHSV field isolate, a 3 amino acid code was attributed based on their sequence, as an *a posteriori* verification of their phenotype. Twenty-seven highly virulent VHSV field strains (all isolated from trout except for one strain isolated from pike), among the 31 included in the phylogeny, had a similar R/KAR code. The majority of low and moderate virulence VHSV isolates (mainly isolated from marine fish) has a genetic code sharing at least one substitution among the three defined in the present study: GAS, RAS, and GAN. Two isolates harbor a unique code: the genotype Ic isolate, DK-3612, with a code EAS corresponding to the unique isolate with a glutamic acid (E) residue in position 46 of the N protein; and the genotype Ia2 isolate, VHSV/O.mykiss/I/TN/480/Oct96, harboring a unique code RER and closely-related to highly-virulent strains. Interestingly, four virus strains isolated from dead rainbow trout and harboring a low-moderate virulence GAS code were found to be highly virulent in rainbow trout with a CPM higher than 42%: the DK-2149, GE-1.2, 2009-50-315-1 and NO-2007-50-385 isolates inducing 66, 53, 79, and 69% of CPM, respectively. In summary, taking into account these few exceptions among the 55 VHSV strains analyzed, the predictive virulence code still leads to a robust correlation with virulence phenotypes in trout in 92.7% of cases.

## Discussion

*Viral hemorrhagic septicemia virus* is economically one of the most important viral disease agents for farmed rainbow trout in Europe. VHSV has become endemic in most of continental Europe since the 1960s. After more than 50 years of surveillance programs the disease was finally eradicated in Denmark in 2009. An eradication plan for VHSV and IHNV is also currently in progress in France. However, the discovery of VHSV-like viruses in many free-living marine fish species in Northern Europe has complicated the situation as marine fish species are potential reservoirs of disease (Skall et al., 2005). Fish farming is characterized by very close contacts between farmed and wild fish and transmission of the virus from fish to fish can occur through the water. The viruses from the two environments (marine and fresh water) are serologically indistinguishable, but under experimental conditions marine isolates have often shown to be non-virulent or of low-pathogenicity to rainbow trout (Skall et al., 2004). However, when introduced into trout farms they can become pathogenic and further increase their virulence over time under intensive aquaculture conditions. Understanding the mechanism of virus evolution toward increased virulence in the aquaculture environment is a requirement for being able to take appropriate preventive actions. Reverse genetics systems allowing manipulation of the viral genome and recovery of live recombinant viruses are available for several VHSV strains (Biacchesi et al., 2010; Biacchesi, 2011; Kim et al., 2014; Yusuff et al., 2019). Thus, it is now relatively easy to generate recombinant viruses harboring targeted genome mutations and to test their impact on virulence. Several genome sequences from marine and freshwater VHSV isolates displaying varying levels of virulence in rainbow trout were determined these past few years. From the analysis of these sequences, several groups have already identified putative amino acid residues that could be involved in VHSV strain virulence (Betts and Stone, 2000; Campbell et al., 2009; Ito et al., 2016, 2018). Interestingly, these authors pointed out that as few as 4–10 amino acid changes were identically substituted between marine and freshwater strains suggesting that only a limited set of amino acid residues might be involved in regulating the level of virulence between isolates. These amino acid substitutions were found in all six viral proteins. Using reverse genetics systems, several groups either exchanged entire ORFs between low- and high-virulence strains (Einer-Jensen et al., 2014; Vakharia et al., 2019; Yusuff et al., 2019) or introduced single amino acid substitutions (Kim et al., 2014; Baillon et al., 2017) in order to determine their biological relevance in VHSV virulence. Several conclusions, sometimes contradictory, were drawn from those studies. Studies based on chimeric recombinant viruses with exchanged G, NV, and L genes did not show any alteration of the virulence phenotype in trout (Einer-Jensen et al., 2014; Yusuff et al., 2019), although another study pointed out that a single mutation in the L protein (F1012I) is possibly involved in host species virulence of various VHSV strains (Kim et al., 2014). More recently, N and P proteins were defined as main determinants responsible for VHSV virulence in trout (Vakharia et al., 2019). These studies were all restricted to the use of only two VHSV strains which were not always closely-related and belonging to different genotypes (genotype I versus genotype IV). In addition, the virulence of recombinant VHSV variants was sometimes only evaluated *in vitro* in cell culture and when tested *in vivo*, the route of infection was limited to the intra-peritoneal (IP) injection, which bypasses natural infection barriers. Thus, it is likely that different mechanisms of virulence might exist and that other viral proteins are actually involved in virulence of VHSV in rainbow trout. In a recent study (Baillon et al., 2017), the sequence comparison of 14 VHSV isolates with different virulence phenotypes in trout, led to the observation of recurrent signatures at one amino acid position of the NV protein (i.e., NV116), that discriminated virulent isolates (harboring R) from attenuated strains originally isolated from non-salmonid species (harboring S or N). The study demonstrated that while the R116N substitution in a recombinant VHSV with a highly virulent backbone had no impact of its *in vivo* phenotype, the R116S substitution resulted in a strong attenuation of virulence. This result highlights the essential role of the NV protein in virulence and that not all VHSV strains followed the same evolutionary pathway toward increased virulence.

The present study is based on the analysis of a larger number of field strains (55 strains) isolated in different fish species which were fully sequenced and phenotyped in trout (Panzarin et al., 2020). This previous analysis pointed out several polymorphisms distributed over the 6 viral proteins and likely involved in different virulence mechanisms. According to the analysis of these 55 genomes, 29 amino acid changes were selected and introduced, alone or in combination, in a highly-virulent VHSV genome backbone by reverse genetics. A total of 35 rVHSV variants were recovered and phenotyped in rainbow trout by bath immersion. These results confirmed the important role in virulence of position 116 at the C-terminal of the NV protein (Figure 5A and Table 1). This position is part of an intrinsically disordered region of the NV protein. Intrinsically disordered regions of multifunctional viral proteins interact with multiple binding partners to accomplish signaling, regulation, and control functions in infected cells (Whelan et al., 2016). Such regions of viral proteins can be involved in loss or gain of function in virus-host interactions, but also represent interesting targets for attenuation of viral replication. Thus, the intrinsically disordered region of the NV protein is likely to bind specific cellular partners, such as those recently published in the NV interactome (Biacchesi et al., 2017). Further studies will be needed to assess the impact of such substitutions in the NV protein function(s), in particular on its efficiency to recruit cellular components to inhibit the antiviral response in different host species.

These data also highlighted a contribution of the G protein in VHSV virulence. Mammalian rhabdovirus glycoproteins are known to play a predominant role in the pathogenesis, and amino acid changes were reported as key determinants for virulence (Ito et al., 2001, 2010; Martinez et al., 2003; Faber et al., 2005). But such demonstrations for fish rhabdoviruses have not been documented yet. The present study highlighted the contribution of the glycoprotein G to the virulence of VHSV. The T135A substitution had a highly attenuating effect both *in vitro* (10-fold reduction in the final titer) and *in vivo* (42% of final survival rate compared to 3% for the parental rVHSV) (Figure 4C and Table 1). The effect of this amino acid substitution will need further investigations *in vivo*, such as studying its effect on viral entry and spread in trout using a non-invasive bioluminescence approach, and on the induced immunogenicity by bath immersion.

Furthermore, this work unraveled a new essential role of N during VHSV infection in trout. Two amino acid changes had a strong effect on virulence when introduced in the highly-virulent rVHSV backbone: K46G and A241E (Figure 2 and Table 1). The A241E substitution was found in a unique strain isolated in rainbow trout from Italy (VHSV/O.mykiss/I/TN/480/Oct96). This strain is closely-related to several highly-virulent strains but displayed only 14% CPM. When inserted in rVHSV 23–75 backbone, this substitution greatly reduced its virulence and yielded a similar CPM of 25% at 30 days post-infection (Figure 2B), indicating that this particular position is largely responsible for the attenuated phenotype of VHSV/O.mykiss/I/TN/480/Oct96. In contrast, the substitution K/R46G is present in several field strains isolated mainly from non-salmonid fish and inducing low to moderate rates of mortality with the exception of four strains all isolated from dead trout (DK-2149, GE-1.2, 2009-50-315-1, and NO-2007-50-385, see below). When this position was modified in the rVHSV genome (K46G), surprising phenotypes were observed both in salmonid-derived cells (Figure 8C; CHSE and Figure 8D; RTG-2) and in trout (Figure 2A). Although rVHSV-N K46G replicated and reached a similar titer in non-salmonid cells such as EPC and BF-2, this variant displayed a delay of approximately 24 h in its replication in salmonid cells, but finally reached a somewhat similar final titer compared to the wild-type rVHSV at 6 days postinfection. In trout, rVHSV-N K46G did not induced any mortality by bath immersion (Figure 2A). In fact, this variant did not establish a productive infection in the fins and was rapidly cleared, as observed with a non-invasive bioluminescence approach (Figures 3A,B). Notably, this variant did not induce any protective immune response in infected fish which died upon a secondary challenge with the wild-type rVHSV (Figure 2C). Even after injection, rVHSV-N K46G was still highly attenuated and only induced 24% of CPM compared to 100% for the parental virus at the lethal dose of 10^6^ PFU/fish (Figure 3C). Position 46 of the N protein of VHSV has already been hypothesized to be involved in virulence (Campbell et al., 2009; Ito et al., 2018; Vakharia et al., 2019), but the present study is the first to demonstrate that this position is clearly a virulence marker. Since the K46G substitution had no effect on the replication in non-salmonid cells and on the final titer in salmonid cells, although viral kinetics were delayed, it could be hypothesized that this position might be involved in protein-protein interactions (PPI) either for the recruitment of a cellular partner for an efficient replication or for the early inhibition of the antiviral response. This is the case for the N protein of rabies virus which was reported to inhibit the RIG-I pathway that controls the expression of interferon (Masatani et al., 2010, 2011). Masatani et al. (2010, 2011) demonstrated that rabies virus N protein mediated the evasion from RIG-I sensing and thus inhibited activation of the transcription factor IRF3. They showed that the amino acids at positions 273 and 394 of the N protein were important to block RIG-I-mediated antiviral response and consequently for rabies virus pathogenicity *in vivo*. This highlights the importance of host innate immunity evasion in viral pathogenicity.

Finally, another virulence marker was confirmed in the polymerase protein L (F1012I) with a less drastic impact but with a pronounced size-dependent effect visible with the increasing size of the animals and the route of infection (bath immersion). This variation in L was already reported as a key amino acid position for *in vitro* virulence of VHSV in rainbow trout gill epithelial cells but the recombinant virus generated by reverse genetics was not tested *in vivo* (Kim et al., 2014). In the present study, rVHSV-L F1012I was tested *in vivo* and did not reveal a marked attenuated phenotype in trout although the kinetics of mortality was significantly slower (Figure 6B). However, this variation in L in combination with another one in the N protein (G392E) led to a more pronounced attenuated phenotype in larger trout, highlighting a potential role of both positions in VHSV virulence in trout (Figure 7B).

The three amino acid code established in the present study was based on the three substitutions with the most pronounced effect on VHSV virulence in rainbow trout. This code was overlaid on the phylogenetic tree generated from a previous genetic and virulence analyses based on 55 VHSV isolates (Panzarin et al., 2020). These three virulence markers explain the 92.7% of the phenotypes observed in rainbow trout for all 55 VHSV strains. For the first time, these data demonstrate the hypothesis initially formulated by Campbell et al. (2009) suggesting that a few amino acid changes can cause significant variation in the virulence phenotype of VHSV isolates. The present study brought a more complete perspective of virulence gain in rainbow trout by VHSV strains. However, as demonstrated here, several viral proteins are responsible for the virulence of VHSV strains and different virulence mechanisms could be involved in different VHSV strains. This is well illustrated by the VHSV/O.mykiss/I/TN/480/Oct96 strain for which only one amino acid change at position 241 of the N seemed to explain its low virulence in trout (CPM of 14%). In contrast, although harboring a GAS genetic code for low/moderate virulence, four exceptions of high virulence in trout were found: DK-2149 (isolated from trout and leading to 53% of CPM), GE_1.2 (isolated from trout and leading to 66% of CPM), 2009-50-315-1 (isolated from trout and leading to 79% of CPM) and NO-2007-50-385 (isolated from trout and leading to 69% of CPM). Those 4 viruses are closely related to low virulence strains. This intriguing observation led to the hypothesis that additional amino acid changes might counteract the effect of the attenuating substitutions K46G in N and R116S in NV. During the construction of the VHSV infectious cDNA harboring the K46G substitution in N, an accidental mutation was introduced in one clone at position 42 of the N, substituting the glycine (G) with an arginine (R). The recombinant virus, rVHSV-N K46G G42R, was readily recovered at a high titer of 2.5 × 10^8^ PFU/mL. When compared to the avirulent rVHSV-N K46G variant, rVHSV-N K46G G42R was virulent in trout by bath immersion with 36% of CPM at day 35 post infection (Supplementary Figure 1A), indicating that the G42R substitution was sufficient to counteract, at least in part, the effect of the K46G substitution. In fact, in the N protein of DK-2149, a lysine (K) is present at the position 43 of the N protein (Supplementary Figure 1B). Positions 42, 43, and 46 on the N protein are in close proximity and arginine and lysine have similar biochemical properties which could explain in part the virulence phenotype of this strain in rainbow trout. It can be hypothesized that other substitutions at different positions and/or in other viral proteins might contribute to increase the virulence of viruses harboring the low/moderate virulence GAS code. This underscores the potential of VHSV for rapid viral evolution as observed for other RNA viruses. The mutation potential could result in the development of resistance to natural antiviral mechanisms and in the possibility of interspecies transmission from wild reservoirs, challenging the management of VHS in farmed and wild fish species.

## Conclusion

This is the first study carried out on such a comprehensive scale of fully sequenced and phenotyped VHSV field isolates which reinforces the characterization of the markers of virulence. These results clearly point out the important roles of the N and NV proteins in VHSV pathogenicity. Concerning the N protein, a previous study pointed out that the decrease of N expression during viral replication has an attenuating effect of IHNV virulence in trout (Rouxel et al., 2016). This was explained by a slowdown in viral replication. But, in view of the results presented here, it can also be hypothesized that other functions of N were affected explaining in part the high induction of the interferon response in infected cells. Thus, the identification of key viral proteins involved in VHSV virulence will lead to the design of safe live-attenuated vaccines against this important pathogen for the trout industry. Finally, this work forms a solid basis to allow the development of diagnostic tools to predict the *in vivo* phenotype of viral field isolates.

## Data Availability Statement

The datasets generated for this study can be found in online repositories. The names of the repository/repositories and accession number(s) can be found in the article.

## Ethics Statement

The animal study was reviewed and approved by the local ethics committee on animal experimentation (COMETHEA INRA no. 45 and ANSES/ENVA/UPC no. 16) and were authorized by the Ministère de l’Éducation nationale, de l’Enseignement supérieur et de la Recherche under the numbers: APAFIS#2545-2015121515466368 v1, APAFIS#2016053117453469, and 08/04/14-10.

## Author Contributions

LB, EM, YB, NO, VP, TM, MB, and SB designed the experiments. LB, EM, JC, LL, EV, and AA conducted the experiments. LB, EM, VP, AC, MB, and SB analyzed the data. AC, VP, MB, and SB wrote the manuscript. All authors have read and approved the final version of the manuscript.

## Conflict of Interest

The authors declare that the research was conducted in the absence of any commercial or financial relationships that could be construed as a potential conflict of interest.
