# Supplementary material for: The Viral Hemorrhagic Septicemia Virus (VHSV) Markers of Virulence in Rainbow Trout (Oncorhynchus mykiss)
Source: Front Microbiol. 2020 Oct 20;11:574231. doi: 10.3389/fmicb.2020.574231 (PMC7606196; doi:10.3389/fmicb.2020.574231)
Supplement: Supplementary file 1 [file Image_1.pdf]

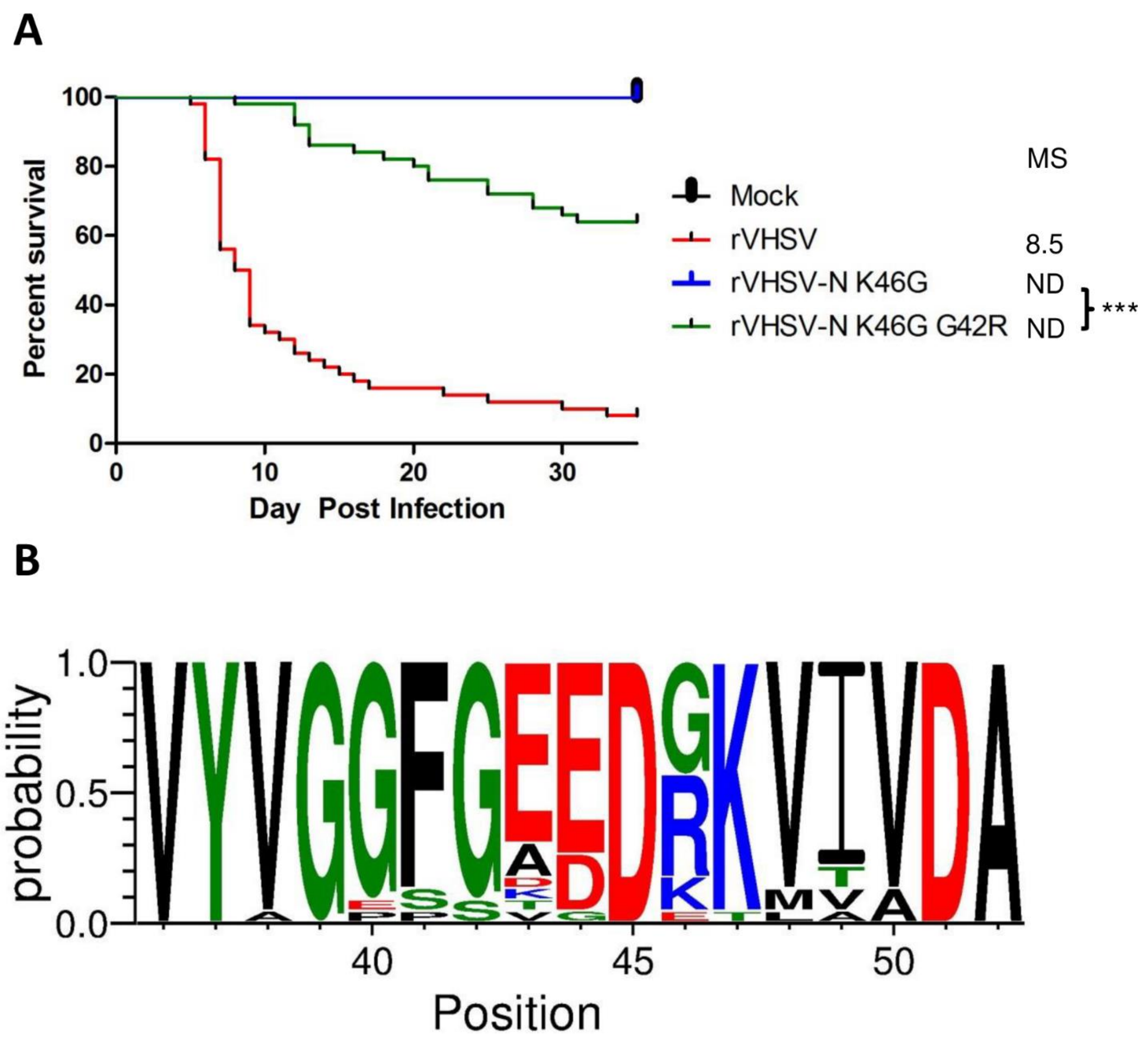

Supplementary Figure S1. Effect of an accidental mutation G42R in N protein on rVHSV-N K46G *in vivo* phenotype

A Juvenile trout (mean weights, 5.3 g; n=50 per groups) were infected by bath immersion with 5 × 10^4 PFU/mL of each of the indicated variants. During the mutagenesis reaction to create the N K46G substitution, an additional G42R mutation was accidentally introduced in the N, leading to rVHSV-N K46G/G42R. Controls are wild-type rVHSV 23-75 (rVHSV) and non-infected trout (mock). Mortality was recorded daily and is presented as percent of survival. Median survival (MS) and statistical significance (*** for p<0.001) are shown on the right of the graph.

B Sequence logo plot representing normalized amino acid frequencies in the N protein domain located between amino acid 36 to 52. Sequences of 23 unique N domains (among the 55 VHSV strains selected in the present study) were analyzed using WebLogo 3.7.3 (http://weblogo.threeplusone.com/; Crooks GE, Hon G, Chandonia JM, Brenner SE WebLogo: A sequence logo generator, Genome Research, 14:1188-1190, (2004)).
